# Delirium-related factors and their prognostic value in patients undergoing craniotomy for brain metastasis

**DOI:** 10.3389/fneur.2022.988293

**Published:** 2022-09-26

**Authors:** Jihwan Yoo, Bio Joo, Juyeong Park, Hun Ho Park, Mina Park, Sung Jun Ahn, Sang Hyun Suh, Jae-Jin Kim, Jooyoung Oh

**Affiliations:** ^1^Department of Neurosurgery, Brain Tumor Center, Gangnam Severance Hospital, Yonsei University College of Medicine, Seoul, South Korea; ^2^Department of Radiology, Gangnam Severance Hospital, Yonsei University College of Medicine, Seoul, South Korea; ^3^Institute of Behavioral Sciences in Medicine, Yonsei University College of Medicine, Seoul, South Korea; ^4^Department of Psychiatry, Gangnam Severance Hospital, Yonsei University College of Medicine, Seoul, South Korea

**Keywords:** delirium, brain metastasis, cancer, postoperative hematoma, Kaplan-Meier survival analysis, craniotomy

## Abstract

**Background:**

Delirium is characterized by acute brain dysfunction. Although delirium significantly affects the quality of life of patients with brain metastases, little is known about delirium in patients who undergo craniotomy for brain metastases. This study aimed to identify the factors influencing the occurrence of delirium following craniotomy for brain metastases and determine its impact on patient prognosis.

**Method:**

A total of 153 patients who underwent craniotomy for brain metastases between March 2013 and December 2020 were evaluated for clinical and radiological factors related to the occurrence of delirium. Statistical analysis was conducted by dividing the patients into two groups based on the presence of delirium, and statistical significance was confirmed by adjusting the clinical characteristics of the patients with brain metastases using propensity score matching (PSM). The effect of delirium on patient survival was subsequently evaluated using Kaplan–Meier analysis.

**Results:**

Of 153 patients, 14 (9.2%) had delirium. Age (*P* = 0.002), sex (*P* = 0.007), and presence of postoperative hematoma (*P* = 0.001) were significantly different between the delirium and non-delirium groups. When the matched patients (14 patients in each group) were compared using PSM, postoperative hematoma showed a statistically significant difference (*P* = 0.036) between the delirium and non-delirium groups. Kaplan–Meier survival analysis revealed that the delirium group had poorer prognosis (log-rank score of 0.0032) than the non-delirium group.

**Conclusion:**

In addition to the previously identified factors, postoperative hematoma was identified as a strong predictor of postoperative delirium. Also, the negative impact of delirium on patient prognosis including low survival rate was confirmed.

## Introduction

Delirium is an acute neuropsychiatric syndrome characterized by an altered mental state, sudden fluctuations in consciousness and cognition, and impaired attention. Various factors have been reported to be associated with the occurrence of delirium, including old age, male sex, reduced cognitive function, electrolyte abnormalities, infection, use of certain medications such as benzodiazepine, and hypoxia (1–5). Although delirium may resolve within a few days, it can result in adverse outcomes, including increased mortality, functional decline, prolonged hospitalization, increased healthcare costs, and long-term cognitive consequences (6). Therefore, recognizing and mitigating the risk factors for delirium is an important strategy in the clinical setting (7).

Brain metastases are the most frequent intracranial tumors in adults, outnumbering primary brain tumors (8). They can cause various neurological dysfunctions, including seizures, paralysis, language deficits, and cognitive decline, which negatively affect the quality of life of the patients (8). In this therapeutic scenario, surgical resection is crucial for establishing a pathological diagnosis, reducing mass effects, and consequently, enhancing neurological function and quality of life in patients with brain metastases (9). However, postoperative delirium has been reported to be prevalent in patients after intracranial surgery, with an incidence of 4.2 to 23% (2–4, 10–13). Although several studies have investigated the risk factors for postoperative delirium after craniotomy, little is known about the factors associated with postoperative delirium in patients who have undergone craniotomy for brain metastases.

Therefore, we aimed to identify the clinical and radiological factors influencing the occurrence of postoperative delirium following craniotomy for brain metastases, focusing on preoperative and immediate postoperative images. We also aimed to determine the impact of postoperative delirium on the prognosis of patients after craniotomy for brain metastases.

## Methods

### Participants

**Figure 1:**
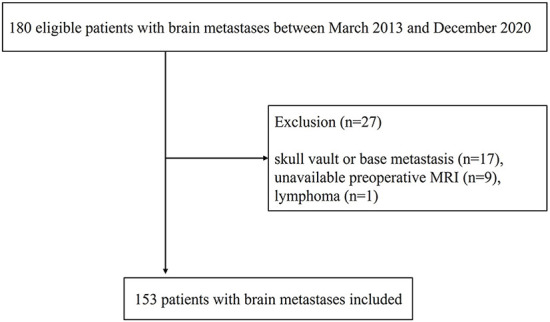
The flowchart of this study.

This retrospective study was conducted with institutional review board approval (3-2022-0168, Gangnam Severance Hospital), and the requirement for patient consent was waived. We retrospectively searched the electronic medical records and identified patients who underwent craniotomy for brain metastases between March 2013 and December 2020. Among the 180 confirmed cases, patients with bone metastasis, such as skull vault or base metastasis (*n* = 17), unavailable preoperative magnetic resonance imaging (MRI) images (*n* = 9), and pathologies other than brain metastasis (*n* = 1) were excluded. Finally, 153 patients with brain metastases were included in this study (Figure 1). Clinical data were anonymized and processed according to the institutional guidelines. Clinical variables that may affect the prognosis of patients prior to craniotomy, particularly those that comprise a graded prognostic assessment (GPA) (age, Karnofsky performance score [KPS], number of brain metastases, and presence of extracranial metastasis) were recorded, along with observable radiological parameters (14). Postoperative laboratory results, pain, and blood pressure (BP) data were acquired on the first postoperative day.

### Treatment

For brain metastases with neurological symptoms or those detected through response assessment, the treatment method was determined according to age, performance status, primary cancer status, neurological symptoms, and size, number, and location of metastases. The craniotomy indications were the same as those previously reported (15). Gross total resection was achieved by postoperative MRI of all tumors, for which craniotomy was planned. In Table 1, residual tumors are defined as cases in which multiple tumors existed prior to craniotomy, and tumors remained outside the surgical site on postoperative MRI.

**Table 1:** Demographics of all cohorts (*N* = 153).

**Delirium**	**Non-delirium (*N* = 139)**	**Delirium (*N* = 14)**	**Total (*N* = 153)**	***p***
**Sex**				
F	78 (56.1%)	2 (14.3%)	80 (52.3%)	0.007
M	61 (43.9%)	12 (85.7%)	73 (47.7%)	
Age	57.6 ± 11.0	67.4 ± 10.5	58.5 ± 11.3	0.002
**HTN**				
No	108 (77.7%)	9 (64.3%)	117 (76.5%)	0.425
Yes	31 (22.3%)	5 (35.7%)	36 (23.5%)	
**DM**				
No	121 (87.1%)	13 (92.9%)	134 (87.6%)	0.839
Yes	18 (12.9%)	1 (7.1%)	19 (12.4%)	
**Preoperative neurologic symptom**				
No	60 (43.2%)	7 (50.0%)	67 (43.8%)	0.835
Yes	79 (56.8%)	7 (50.0%)	86 (56.2%)	
**Preoperative KPS**				
90–100	25 (18.0%)	1 (7.1%)	26 (17.0%)	0.515
70–80	94 (67.6%)	10 (71.4%)	104 (68.0%)	
< 70	20 (14.4%)	3 (21.4%)	23 (15.0%)	
**Postoperative KPS**				
90–100	50 (36.0%)	3 (21.4%)	53 (34.6%)	0.070
70–80	71 (51.1%)	6 (42.9%)	77 (50.3%)	
< 70	18 (12.9%)	5 (35.7%)	23 (15.0%)	
**Extracranial metastasis**				
No	67 (48.2%)	9 (64.3%)	76 (49.6%)	0.386
Yes	72 (51.8%)	5 (35.7%)	77 (50.3%)	
**Primary cancer**				
Breast	36 (25.9%)	0 (0.0%)	36 (23.5%)	0.184
GI	16 (11.5%)	4 (28.6%)	20 (13.1%)	
GU	9 (6.5%)	1 (7.1%)	10 (6.5%)	
Gyn	7 (5.0%)	0 (0.0%)	7 (4.6%)	
HCC	7 (5.0%)	0 (0.0%)	7 (4.6%)	
Lung	58 (41.7%)	8 (57.1%)	66 (43.1%)	
Melanoma	3 (2.2%)	0 (0.0%)	3 (2.0%)	
Thyroid	3 (2.2%)	1 (7.1%)	4 (2.6%)	
**Multiplicity**				
No	77 (55.4%)	6 (42.9%)	83 (54.2%)	0.538
Yes	62 (44.6%)	8 (57.1%)	70 (45.8%)	
**Multi-lobe involvement**				
No	83 (59.7%)	6 (42.9%)	89 (58.2%)	0.350
Yes	56 (40.3%)	8 (57.1%)	64 (41.8%)	
**Leptomeningeal seeding**				
No	133 (95.7%)	14 (100.0%)	147 (96.1%)	0.944
Yes	6 (4.3%)	0 (0.0%)	6 (3.9%)	
**No. of BM**				
1	77 (55.4%)	6 (42.9%)	83 (54.2%)	0.510
2–3	31 (22.3%)	5 (35.7%)	36 (23.5%)	
>3	31 (22.3%)	3 (21.4%)	34 (22.2%)	
**Tumor location**				
Cbll	33 (23.7%)	8 (57.1%)	41 (26.8%)	0.091
F	40 (28.8%)	3 (21.4%)	43 (28.1%)	
O	12 (8.6%)	0 (0.0%)	12 (7.8%)	
P	44 (31.7%)	2 (14.3%)	46 (30.1%)	
T	10 (7.2%)	1 (7.1%)	11 (7.2%)	
**Preoperative peritumoral edema**				
No	3 (2.2%)	1 (7.1%)	4 (2.6%)	0.814
Yes	136 (97.8%)	13 (92.9%)	149 (97.4%)	
Tumor volume (ml)	19.5 ± 24.4	16.1 ± 8.4	19.2 ± 23.4	0.266
**Intratumoral necrosis**				
No	101 (72.7%)	8 (57.1%)	109 (71.2%)	0.361
Yes	38 (27.3%)	6 (42.9%)	44 (28.8%)	
**Intratumoral hemorrhage**				
No	123 (88.5%)	14 (100.0%)	137 (89.5%)	0.377
Yes	16 (11.5%)	0 (0.0%)	16 (10.5%)	
**Postoperative hematoma**				
No	121 (87.1%)	7 (50.0%)	128 (83.7%)	0.001
Yes	18 (12.9%)	7 (50.0%)	25 (16.3%)	
**Postoperative extracavity hematoma**				
No	136 (97.8%)	13 (92.9%)	149 (97.4%)	0.814
Yes	3 (2.2%)	1 (7.1%)	4 (2.6%)	
**Residual tumor**				
No	79 (56.8%)	6 (42.9%)	85 (55.6%)	0.471
Yes	60 (43.2%)	8 (57.1%)	68 (44.4%)	
Postoperative systolic BP (mmHg)	154.9 ± 20.5	169.6 ± 21.1	156.3 ± 20.9	0.012
Postoperative diastolic BP (mmHg)	93.6 ± 11.0	93.4 ± 16.4	93.6 ± 11.5	0.968
Postoperative pain (VAS)	3.6 ± 2.5	3.6 ± 2.4	3.6 ± 2.5	0.924
Postoperative glucose (mg/dL)	139.2 ± 39.8	143.2 ± 32.8	139.5 ± 39.1	0.707
Postoperative Na (mmol/L)	141.0 ± 3.8	139.2 ± 3.0	140.8 ± 3.8	0.094
Postoperative K (mmol/L)	4.0 ± 0.4	4.3 ± 0.4	4.0 ± 0.4	0.008
Postoperative Cl (mmol/L)	108.9 ± 3.5	108.4 ± 2.3	108.8 ± 3.4	0.637

HTN, hypertension; DM, Diabetus Mellitus; KPS, Karnofsky performance score; GI, gastrointestinal; GU, genitourinary; Gyn, gynecologic; HCC, hepatocellular carcinoma; BM, brain metastases; Cbll, cerebellum; F, frontal; O, occipital; P, parietal; T, temporal; BP, blood pressure; VAS, visual analog scale.

After craniotomy, all patients received whole-brain radiation therapy (WBRT), which was administered at a total dose of 30 Gy in ten fractions. After completion of WBRT, systemic chemotherapy (including targeted therapy and immunotherapy) was implemented considering each primary cancer type, genotype, and previous treatment.

### Image acquisition and assessment

All patients routinely underwent preoperative non-enhanced brain computed tomography (CT) and contrast-enhanced MRI one week before surgery, postoperative non-enhanced brain CT scan on the day of surgery, and postoperative contrast-enhanced MR within 24 h after surgery. Additional imaging was performed if necessary. All CT scans were performed using a 128-channel CT scanner (SOMATOM Definition AS+; Siemens, Germany) with the following parameters: tube voltage of 100 kVp, 260 mAs, 512 × 512 matrix, 240 mm Field-of-view (FOV), and 4 mm slice thickness. Another set of axial, coronal, and sagittal images were reconstructed at a thickness of 1 mm. MRI was performed using a 3T scanner (Discovery MR750; GE Healthcare, United States) with a 16-channel head coil, including axial T2-weighted imaging, non-enhanced sagittal 3D T1-weighted imaging, sagittal 3D Fluid attenuated inversion recovery (FLAIR), axial 3D susceptibility-weighted imaging, and contrast-enhanced sagittal 3D T1-weighted imaging. The imaging parameters for MRI were as follows: 1) axial T2-weighted imaging: repetition time/echo time (TR/TE), 5320/102 ms; flip angle, 142°; section thickness, 4 mm; FOV, 230 mm; matrix, 352 × 352. 2) non-enhanced sagittal 3D T1-weighted imaging: TR/TE, 8.2/3.2 ms; flip angle, 12°; section thickness, 1 mm; FOV, 240 mm; matrix, 256 × 256. 3) Sagittal 3D FLAIR imaging: TR/TE, 6000/89 ms; TI, 1741 ms; section thickness, 1.2 mm; FOV, 260 mm; matrix, 256 × 224. 4) Axial 3D susceptibility-weighted imaging: TR/TE, 30.9/23.4 ms, 46.8 ms, and 70.2 ms; flip angle, 10°; section thickness, 2 mm; FOV, 230 mm; matrix, 320 × 224. 5) Contrast-enhanced sagittal 3D T1-weighted imaging: TR/TE, 8.2/3.2 ms; flip angle, 12°; section thickness, 1 mm; FOV, 230 mm; matrix, 256 × 256.

Two neuroradiologists with 3 and 9 years of experience in neuroradiology, who were blinded to the clinical data, independently assessed the number of brain metastases, multi-lobe involvement, resected tumor location, and presence of leptomeningeal seeding, peritumoral edema, and intratumoral necrosis and hemorrhage on preoperative MRI. In addition, the presences of postoperative intracranial hemorrhage were assessed using postoperative CT and MRI. Postoperative hemorrhage was defined as a space-occupying hematoma that developed at the tumor resection site, the presence of a characteristic fluid-fluid level at the tumor resection site, or hemorrhage beyond the resection site causing subarachnoid hemorrhage. Minimal residual blood at the tumor resection site was not considered postoperative hemorrhage. Additionally, hemorrhages beyond the resection site were counted separately. This process was conducted with consensus of two neuroradiologists. To measure the total volume of the metastatic tumors, tumor segmentation and active contour segmentation processes were performed on preoperative contrast-enhanced 3D T1-weighted imaging using the open-source software ITK-Snap version 3.8.0 (www.itksnap.org) by another neuroradiologist with 11 years of experience.

### Diagnosis and management of delirium

During routine clinical process, we documented any abnormal cognition or behavior recognized by clinicians after craniotomy. These abnormalities included inattention, aggressive behavior, irrelevant speech, disorientation, line removal, and visual hallucinations. When these possible delirium symptoms occurred, a referral was made to a psychiatrist and he/she confirmed the clinical diagnosis of delirium, and if needed, possible interventions, including administration of antipsychotics and removal of offending drugs, were done after medical evaluation. Delayed onset of delirium in discharged patients after craniotomy, especially those who developed delirium during radiation therapy, the occurrence of delirium was initially determined solely on the basis of patients' or their surrogates' reports; then patients were further referred to a psychiatrist for accurate evaluation of delirium. Because of the retrospective nature of this study, objective indicators such as confusion assessment method (CAM) (16), confusion assessment method for the intensive care unit (CAM-ICU) (17), and clinical rating scale for delirium severity or motor subtypes were omitted.

### Statistical analysis

First, differences in each variable according to the presence or absence of delirium were investigated. For continuous variables, Student's *t*-test was used, and for categorical variables, the chi-squared or Fisher's exact test was used. Propensity score matching (PSM) was used to correct the imbalance of items showing differences between the two groups; we implemented a 1:2 nearest neighbor analysis, with a caliper width of 0.2 standard deviations of the logit distance measured using the R-package, “MatchIt.” Covariates used for matching included age, sex, primary cancer type, and tumor location. Overall survival (OS) was defined as the time from craniotomy for brain metastases to death. Using Kaplan–Meier survival analysis, the effect of delirium occurrence on survival in the two PSM-corrected groups was evaluated. Statistical differences in survival between the two groups were compared using the log-rank test. All statistical analyses were performed using the R software version 4.1.3 (R Foundation for Statistical Computing, Vienna, Austria). Statistical significance was set at *P* < 0.05.

## Results

### Patient characteristics

Of the 153 patients, 14 had delirium and 139 did not. The delirium and non-delirium groups did not differ in most clinical and radiological characteristics, and the only statistically significant variables were age, sex, and postoperative hematoma (Table 1). The presence of neurological symptoms before craniotomy and the preoperative KPS did not differ between the groups. In particular, postoperative KPS tended to be lower in the delirium group than in the non-delirium group, but the difference was not statistically significant (*P* = 0.070). Extracranial metastasis and the number of brain metastases, which consisted of the GPA, did not show any difference according to the occurrence of delirium.

**Figure 2:**
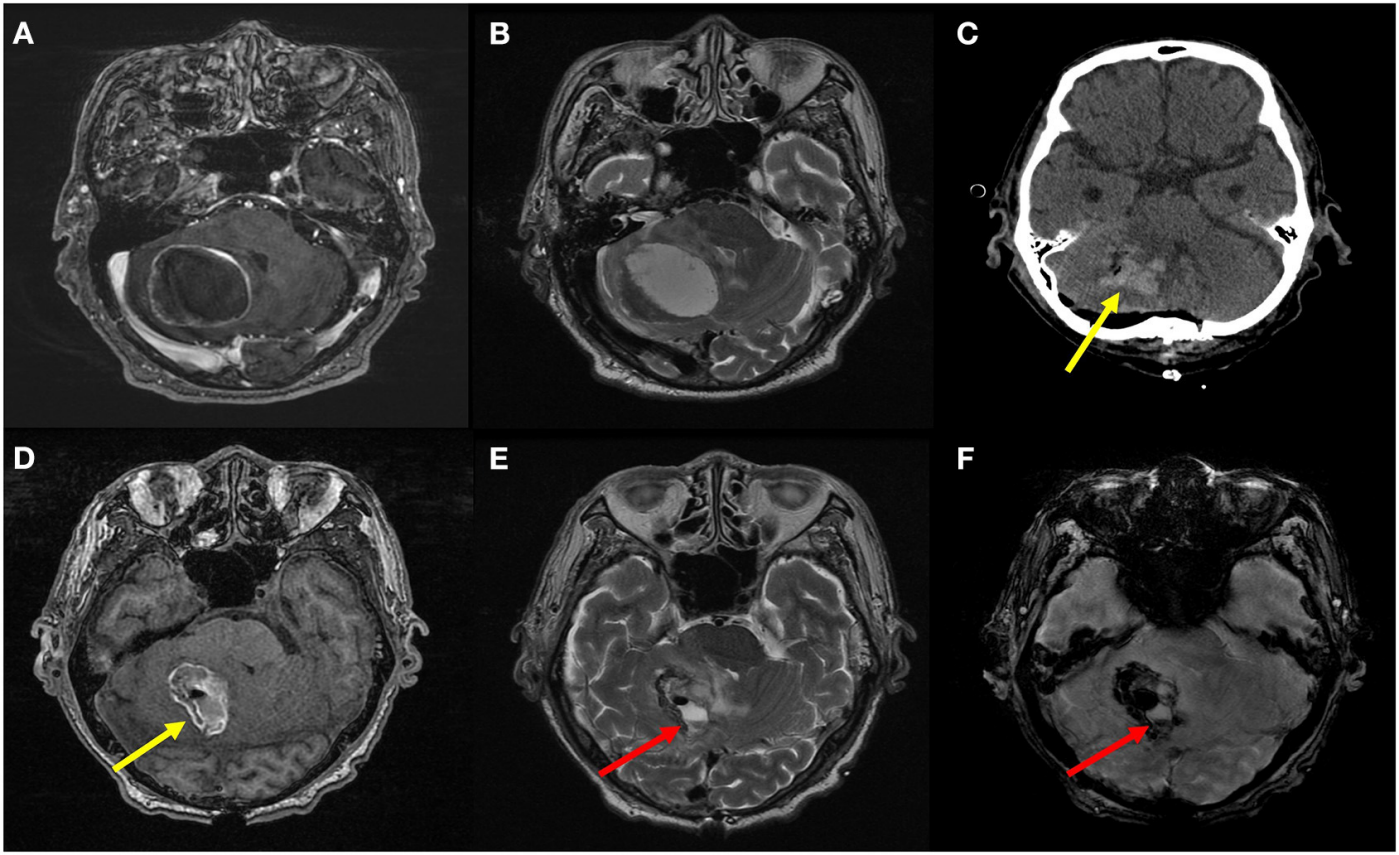
Preoperative T1 contrast-enhanced **(A)** and T2-weighted MR images **(B)** of an 84-year-old male patient with advanced gastric cancer show a well-defined metastatic tumor in the right cerebellar hemisphere with peritumoral edema. On postoperative CT image **(C)** and non-enhanced T1-weighted **(D)**, T2-weighted **(E)**, and susceptibility-weighted **(F)** MR images, space-occupying hematoma (yellow arrows) as well as air bubbles are seen in the resection cavity. Furthermore, a fluid-fluid level (red arrows) is also seen in the resection cavity, which indicates hematoma formation in this context. This was considered as presence of postoperative intracranial hemorrhage. The patient experienced postoperative delirium on the third day after surgery. The motor subtype was non-hypoactive (i.e., hyperactive or mixed); hence, we observed sleep disturbance, irrelevant speech, delusion, and aggressive behavior. The sleep and behavioral problems were managed using several medications: chlorpromazine, risperidone, and trazodone.

**Figure 3:**
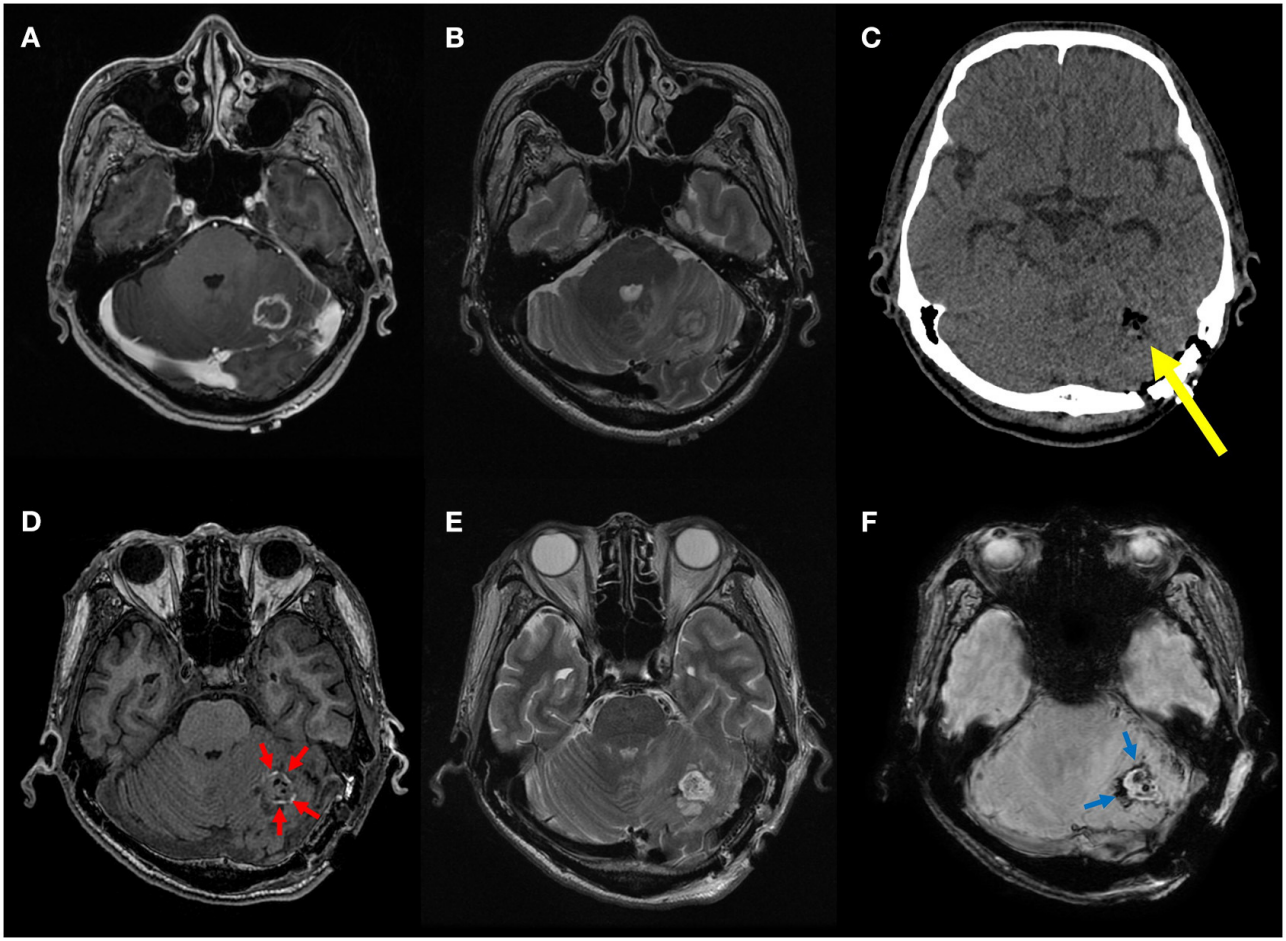
A representative case of minimal residual blood at the resection site. Preoperative T1 contrast-enhanced **(A)** and T2-weighted MR images **(B)** of a 62-year-old male patient with non-small cell lung cancer show a small rim-enhancing metastatic tumor in the left cerebellar hemisphere with peritumoral edema. On postoperative CT image **(C)**, only air bubbles are seen in the resection cavity without discernible hemorrhage (yellow arrow). On postoperative MR images, only thin T1 hyperintense (red arrows) and T2* hypointense rim (blue arrows) is seen along the resection margin without space-occupying hematoma on T1-weighted **(D)**, T2-weighted **(E)**, and susceptibility-weighted images **(F)**. This was considered absence of postoperative intracranial hemorrhage, and the patient did not experience postoperative delirium.

Preoperative radiological features that are important to neurosurgeons, such as peritumoral edema and intratumoral necrosis and hemorrhage, did not differ between the two groups. There was no difference in the presence of residual tumor (*P* = 0.471); however, there was a clear difference between the two groups in the case of postoperative hematoma (*P* = 0.001). In the delirium group, postoperative hematoma was observed in half (7/14, 50.0%) of the patients, which was higher than that in the non-delirium group (18/121, 12.9%). Representative cases with and without postoperative hematoma are shown in Figures 2, 3, respectively.

### Propensity score matching

As the two groups differed in size and reported differences in multiple variables, PSM was used to create two groups, and then the difference in postoperative hematoma was evaluated. Statistical analysis was performed on the group of patients with delirium (*n* = 14) and the group without delirium (*n* = 14), extracted using PSM (Table 2). Gender, age, postoperative systolic blood pressure, and postoperative potassium level, which were statistically different between the two groups before PSM, showed no statistical difference after PSM. Postoperative hematoma was observed in one (7.1%) patient in the non-delirium group and seven (50%) patients in the delirium group, and a statistically significant difference was found (*P* = 0.036). A total of two (14.3%) and eight (57.1%) patients in the non-delirium and delirium groups, respectively, were found to have residual tumors. Before PSM, no statistically significant difference was observed; however, after PSM, a statistically significant difference was observed (*P* = 0.049) in the presence of residual tumors between the groups.

**Table 2:** Propensity score matching (age, sex, primary cancer, tumor location).

**Delirium**	**No delirium**	**Delirium**	**p**
	**(*N* = 14)**	**(*N* = 14)**	
**Sex**			
F	2 (14.3%)	2 (14.3%)	1.000
M	12 (85.7%)	12 (85.7%)	
Age	67.6 ± 5.5	67.4 ± 10.5	0.947
**pre_KPS**			
< 70	1 (7.1%)	3 (21.4%)	0.501
70, 80	11 (78.6%)	10 (71.4%)	
90, 100	2 (14.3%)	1 (7.1%)	
**post_KPS**			
< 70	1 (7.1%)	5 (35.7%)	0.178
70, 80	8 (57.1%)	6 (42.9%)	
90, 100	5 (35.7%)	3 (21.4%)	
**ECM**			
No	7 (50.0%)	9 (64.3%)	0.703
Yes	7 (50.0%)	5 (35.7%)	
**Primary_organ**			
GI	1 (7.1%)	4 (28.6%)	0.518
GU	1 (7.1%)	1 (7.1%)	
Lung	11 (78.6%)	8 (57.1%)	
Thyroid	1 (7.1%)	1 (7.1%)	
**Tumor location**			
Cbll	8 (57.1%)	8 (57.1%)	0.767
F	4 (28.6%)	3 (21.4%)	
P	2 (14.3%)	2 (14.3%)	
T	0 (0.0%)	1 (7.1%)	
**No. of BM**			
1	12 (85.7%)	6 (42.9%)	0.059
2, 3	1 (7.1%)	5 (35.7%)	
>3	1 (7.1%)	3 (21.4%)	
Postoperative systolic BP (mmHg)	156.7 ± 24.8	169.6 ± 21.1	0.152
Postoperative pain (VAS)	4.0 ± 2.6	3.6 ± 2.4	0.711
Postoperative glucose (mg/dL)	146.9 ± 54.7	143.3 ± 32.8	0.833
Postoperative Na (mmol/L)	140.1 ± 3.1	139.2 ± 3.0	0.469
Postoperative K (mmol/L)	4.0 ± 0.4	4.3 ± 0.4	0.091
**Postoperative hematoma**			
No	13 (92.9%)	7 (50.0%)	0.036
Yes	1 (7.1%)	7 (50.0%)	
**Residual tumor**			
No	12 (85.7%)	6 (42.9%)	0.049
Yes	2 (14.3%)	8 (57.1%)	

KPS, Karnofsky performance score; ECM, extracranial metastasis; GI, gastrointestinal; GU, genitourinary; Cbll, cerebellum; F, frontal; O, occipital; P, parietal; T, temporal; BM, brain metastases; BP, blood pressure; VAS, visual analog scale.

**Figure 4:**
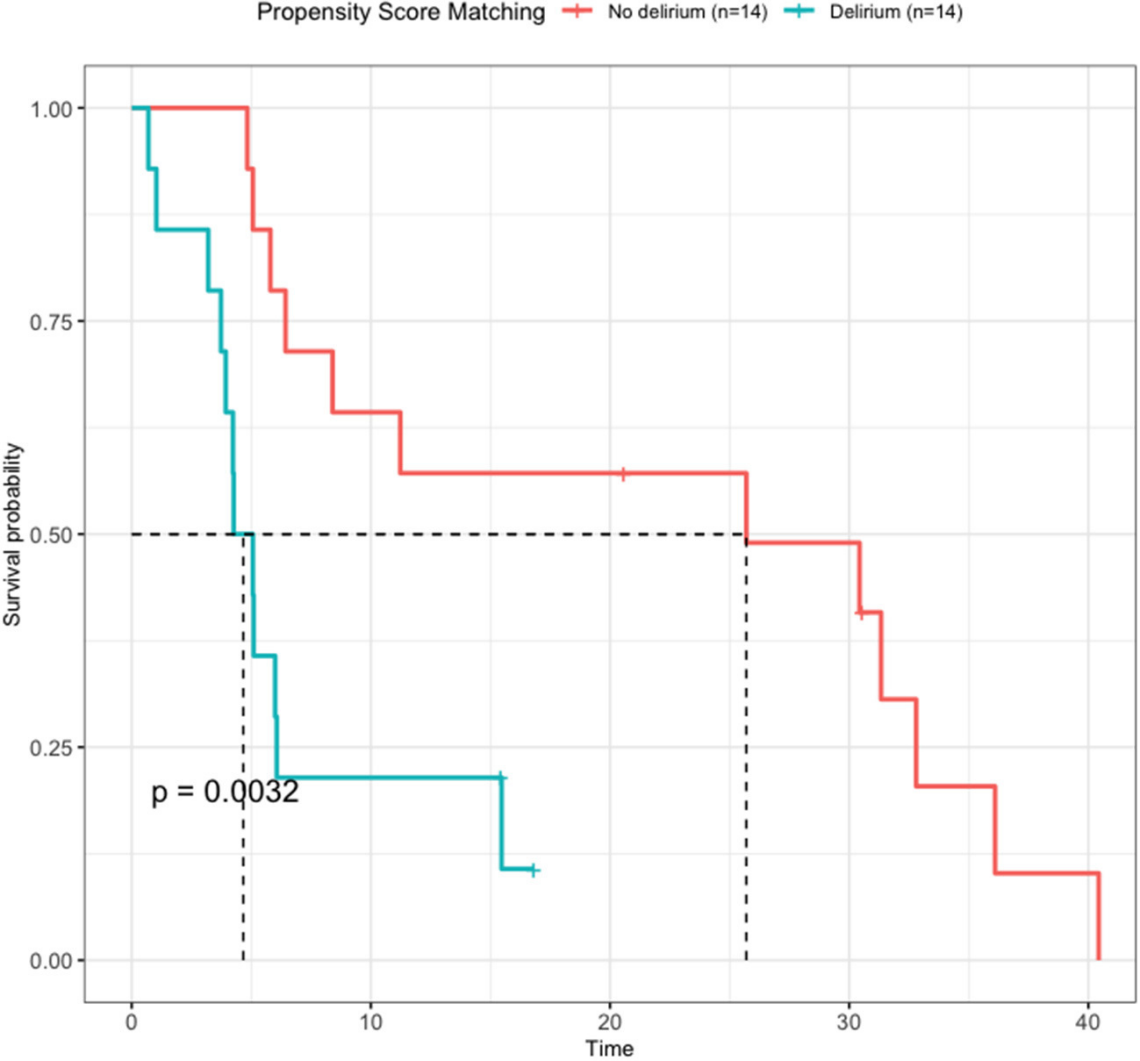
Survival analysis by delirium after propensity score matching. The non-delirium group had a median survival of 25.7 months, while the delirium group had a median survival of 4.67 months (*P* = 0.032).

The OS of the two groups obtained through PSM was compared using Kaplan–Meier survival analysis (Figure 4). The non-delirium group had a median survival of 25.7 months, whereas the delirium group had a median survival of 4.67 months. The log-rank score between the two groups was 0.0032, indicating that delirium affected survival negatively.

### Detailed cognitive and behavioral symptom profile of patients with delirium

Delirium was identified and treated in 14 patients, including eight with lung cancer, four with gastrointestinal cancer, one with genitourinary cancer, and one with thyroid cancer (Table 3). The most frequent time point of occurrence of delirium was the first day after craniotomy, which was observed in seven patients (50.0%). A total of 10 patients (71.4%) developed delirium within a week of craniotomy. Delayed onset of delirium, which occurred after a week, was reported in four patients, ranging from 11th to 30th postoperative day. Among them, three patients reported the symptoms approximately 1 month after craniotomy during routine outpatient follow-up, and the exact onset time was unknown. The most common delirium symptom was aggressive behavior, which was observed in seven patients (50.0%), followed by irrelevant speech in six patients (42.9%). Non-hypoactive type (i.e., hyperactive or mixed-type delirium) was observed in 12 patients (85.7%), and medication was administered to 10 patients (83.3%).

**Table 3:** Clinical features and treatment in patients with delirium.

**Pt #**	**Sex**	**Age**	**Chief complaint**	**Location**	**Primary cancer**	**Onset**	**Medication for sleep disturbance or behavioral problems**	**Motor type**	**Concomitant symptoms**
1	F	69	paraparesis	Cbll	Lung	POD#1	No medication	Hypoactive	Reduced motor activity Drowsiness
2	M	64	Headache	Cbll	Lung	POD#1	Risperidone 0.5 mg	Non-hypoactive	Aggressive Behavior
3	M	71	Gait disturbance	Cbll	Lung	POD#1	Haloperidol PO 1.5 mg	Non-hypoactive	Aggressive Behavior
4	M	71	Lt weakness	Frontal	Lung	POD#1	Risperidone 1 mg	Non-hypoactive	Irrelevant Speech Wandering
5	M	72	Dizziness	Frontal	Thyroid	POD#1	No medication	Non-hypoactive	Slight behavioral problem
6	M	59	Headache	Cbll	GI	POD#1	Haloperidol injection 5 mg Trazodone 25 mg Risperidone 1 mg	Non-hypoactive	Irrelevant Speech Aggressive Behavior
7	M	66	Rt weakness	Cbll	GU	POD#1	Quetiapine 12.5 mg Risperidone 1 mg Haloperidol injection 2.5 mg	Non-hypoactive	IV line removal
8	M	90	Lt weakness	Frontal	GI	POD#2	Risperidone 0.25–1 mg	Non-hypoactive	Aggressive Behavior
9	M	84	General weakness	Cbll	GI	POD#3	Chlorpromazine 50 mg Risperidone 1 mg Trazodone 25 mg	Non-hypoactive	Delusion, Irrelevant Speech Aggressive Behavior
10	F	60	Headache	Temporal	GI	POD#7	Risperidone 0.5 mg	Non-hypoactive	V/H IV line removal
11	M	67	Rt weakness	Cbll	Lung	POD#11	Risperidone 1.5 mg	Non-hypoactive	IV line removal
12	M	48	Headache	Cbll	Lung	POD#30	Quetiapine 12.5–25 mg	Hypoactive	Reduced motor activity Drowsiness Suspicious V/H Irrelevant Speech
13	M	59	Lt weakness	Parietal	Lung	POD#30	Quetiapine 12.5 mg Risperidone 1 mg	Non-hypoactive	Irrelevant Speech Aggressive Behavior
14	M	64	Headache	Parietal	Lung	POD#30	No medication	Non-hypoactive	Memory Impairment V/H Delusion Irrelevant Speech Aggressive Behavior

Cbll, cerebellum; POD, postoperative day; GI, gastrointestinal; GU, genitourinary; V/H, visual hallucination.

## Discussion

In this study, we aimed to identify the factors influencing the development of postoperative delirium in patients who underwent craniotomy for metastatic brain tumors and to elucidate the clinical significance of delirium. Careful postoperative image review revealed that the presence of postoperative hematoma showed a significant difference depending on the occurrence of delirium. In addition, even when the biases of the variables were controlled using statistical techniques, it was discovered that the presence of postoperative hematoma after craniotomy had a significant relationship with the occurrence of delirium. The delirium risk factors reported in previous literature, such as male gender, old age, postoperative hypertension, and electrolyte imbalance, exhibited a statistical significance in the delirium group, but there was no difference after PSM (18–21). This should be interpreted as statistically emphasizing the significance of postoperative hematoma rather than stating that the variables listed above are statistically insignificant. Furthermore, postoperative delirium is a predictor of poor survival outcomes in individuals with metastatic brain tumors. Our findings highlight the clinical significance of postoperative delirium and the need for postoperative imaging.

In general, the risk factors of delirium can be categorized into baseline type (less modifiable; e.g., age, sex, dementia, and surgery) and precipitant type (more modifiable; e.g., medications, infections, and abnormality in laboratory findings) (22). Previous studies have shown that old age and male gender are risk factors for postoperative delirium (10, 11, 23). In patients with brain metastases, delirium can result from the direct effects of metastatic brain regions on the central nervous system or indirect effects, including medications, infections, vascular complications, electrolyte imbalance, and paraneoplastic syndromes (24–27). It should be noted that most brain metastasis-specific factors, such as tumor location, volume, and multiplicity, multilobe involvement, and presence of leptomeningeal seeding and intratumoral hematoma or necrosis, seem to be associated with direct effects. However, most of the previous evidence on causative factors for occurrence of delirium focuses on the indirect effects of brain metastases because usual delirium studies exclude patients with brain tumors or metastases; thus, the direct effects have not yet been sufficiently investigated. The result of the present study which suggests that postoperative intracranial hemorrhage is closely related to the development of delirium may be an opportunity to re-evaluate the importance of the direct regional effect of brain metastases in the development of delirium.

Postoperative intracranial hemorrhage is one of the most serious operation-related conditions following intracranial procedures, and its incidence has been reported to be 10.8–50.0% based on routine radiological monitoring (28). Although its association with significant morbidity and mortality is well known, little is known about the relationship between postoperative intracranial hemorrhage and postoperative delirium occurrence. The definition of postoperative intracranial hemorrhage may be unclear because residual blood is expected in most cases after intracranial intervention (28). Several previous studies have defined significant postoperative intracranial hemorrhage as a hematoma that clinically requires surgical evacuation (29–32). However, in this study, we defined the presence of postoperative hemorrhage as a space-occupying hematoma that developed at a tumor resection site or hemorrhage beyond the resection site, causing subarachnoid hemorrhage on postoperative CT or MRI done within 24 h after tumor resection. This postoperative hemorrhage was distinct from the minimal residual blood at the resection site, which was demonstrated as a thin hypointensity layer on susceptibility-weighted images and a thin hyperdense layer on CT images along the resection margin.

According to our results, the location of the tumor exhibited marginal significance, and patients with cerebellar metastases appeared to be more likely to develop delirium. This finding is in line with previous evidence, which revealed that diffusion tensor imaging abnormality of the cerebellum was the most significant risk factor for postoperative delirium (33). Metastatic tumors in the cerebellum can result in cerebellar cognitive affective syndrome, which is characterized by an impaired ability to regulate or modulate cognition and behavior in a manner that is appropriate to the context. However, further studies with a larger number of patients are needed to clarify the relationship between the location of the tumor resection site and occurrence of postoperative delirium.

Given that the influence of occurrence of postoperative hematoma at the resection site on delirium was the most notable finding regardless of the location, the effect of hematoma itself should be the focus. Delirium is known to be closely related to neuroinflammation, which can be assessed by the increased neutrophil-to-lymphocyte ratio or C-reactive protein level (34). Considering that hematoma can not only cause regional swelling, but also regional febrile and inflammatory responses (35), our findings might be linked to the neuro-inflammation hypothesis of delirium (36). Another important factor that can significantly influence the development of delirium is hypoxic-ischemic injury (37). Intracranial hemorrhage might result in hypoxia in the surrounding neurons, and this mechanism could be another crucial factor in the higher incidence of delirium occurrence in patients with intracranial hemorrhage after neurosurgery.

This study had several limitations. First, the sample size was relatively small compared to the matched sample. Although we tried to use long-term data, the number of patients with delirium included in the study was small compared to other types of clinical samples with a higher incidence of delirium, such as patients who underwent orthopedic or aortic surgery. In addition, this small sample size may have contributed to the increased bias of the PSM. Previous study suggested that PSM can be effective in reducing bias with sample sizes as small as 100 observations for each group (38). Second, our study design was retrospective, and we could not fully evaluate the development of delirium using standardized delirium screening tools. Instead, we retrospectively included patients referred for delirium symptoms. This may have caused under-recognition of the hypoactive subtype, resulting in a small sample size of patients with delirium. Third, this was a single-center study; therefore, careful generalization of our results may be needed. Future prospective studies, which not only assess delirium occurrence more accurately, but also assess the motor subtypes and delirium severity, may address these problems. Although our study may have focused primarily on patients with non-hypoactive subtypes, we revealed various delirium-related factors in patients who underwent craniotomy for brain metastases using matched samples.

To our knowledge, this is the first study to evaluate the effect of delirium development on survival in patients who underwent craniotomy for brain metastases. In our study, the delirium group had a higher frequency of patients with old age, male sex, and postoperative hematoma formation than the non-delirium group. We also found that the occurrence of delirium was associated with significantly low survival rate of the patients. Therefore, the presence of delirium should be regarded as clinically significant when treating patients who underwent craniotomy for brain metastases.

## Data availability statement

The datasets presented in this article are not readily available because data sharing requires permission from the author's institution. Requests to access the datasets should be directed to JO, ojuojuoju@yuhs.ac.

## Ethics statement

The studies involving human participants were reviewed and approved by Institutional Review Board of Gangnam Severance Hospital (3-2022-0168). Written informed consent for participation was not required for this study in accordance with the National Legislation and the Institutional Requirements.

## Author contributions

JY, BJ, and JO designed and conceptualized the study, conducted the literature search, interpreted the data, and drafted and revised the manuscript. JY, BJ, HP, MP, SA, SS, J-JK, and JO collected data. JY, BJ, JP, and JO performed the data analysis. All authors have contributed to the manuscript and approved the submitted version.

## Funding

This study was supported by the National Research Foundation of Korea (NRF) grant funded by the Korean government (MSIT) (No. 2020R1C1C1007440).

## Conflict of interest

The authors declare that the research was conducted in the absence of any commercial or financial relationships that could be construed as a potential conflict of interest.

## Publisher's note

All claims expressed in this article are solely those of the authors and do not necessarily represent those of their affiliated organizations, or those of the publisher, the editors and the reviewers. Any product that may be evaluated in this article, or claim that may be made by its manufacturer, is not guaranteed or endorsed by the publisher.
