# Supplementary material for: Delirium-related factors and their prognostic value in patients undergoing craniotomy for brain metastasis
Source: Front Neurol. 2022 Sep 26;13:988293. doi: 10.3389/fneur.2022.988293 (PMC9548882; doi:10.3389/fneur.2022.988293)
Supplement: Supplementary file 1 [file Table_1.DOCX]

|  | Kappa or Intraclass correlation coefficient (95% CI) |
| --- | --- |
| Number of brain metastases | 0.9995 (0.9994 to 0.9997) |
| Multi-lobe involvement | 1.00000 (1.00000 to 1.00000) |
| Resected tumor location | 1.00000 (1.00000 to 1.00000) |
| Leptomeningeal seeding | 1.00000 (1.00000 to 1.00000) |
| Peritumoral edema | 1.00000 (1.00000 to 1.00000) |
| Intratumoral necrosis | 1.00000 (1.00000 to 1.00000) |
| Intratumoral hemorrhage | 1.00000 (1.00000 to 1.00000) |
| Postoperative hemorrhage | 0.95955 (0.90386 to 1.00000) |

We agree that that interobserver agreement analysis is essential in order to properly understand our results. However, because most of the variables were clear-cut and obvious, there wasn't much disagreement between the two neuroradiologists' assessments. The intraclass correlation coefficient was utilized because the "number of brain metastases" was a continuous variable, whereas the Kappa value was used to evaluate the other variables.
